# Anti-freezing supercapacitors using novel choline phosphate aqueous electrolytes

**DOI:** 10.1080/14686996.2026.2688749

**Published:** 2026-06-19

**Authors:** Jan Malczak, Seyed Amirhossein Sanei, Agnieszka Marcinkowska, Piotr Gajewski, Qamar Abbas

**Affiliations:** a Faculty of Chemical Technology, Poznań University of Technology, Poznań, Poland; b Institute for Chemistry and Technology of Materials, Graz University of Technology, Graz, Austria

**Keywords:** Choline phosphate, anti-freezing electrolyte, carbon electrode, electric double-layer, supercapacitor

## Abstract

Conventional aqueous and organic electrolytes often suffer from toxicity, flammability, poor performance at low temperatures, or limited electrochemical stability, motivating the search for environmentally friendly alternatives. Herein, we report a comprehensive study on aqueous choline – phosphate electrolytes based on choline dihydrogen phosphate (CDHP) and mixed choline dihydrogen phosphate and choline hydrogen phosphate (CDHP + CHP) systems as green electrolytes for low-temperature energy storage devices. Both salts were synthesized via neutralization of choline hydroxide with phosphoric acid. Physicochemical analysis revealed that pure CDHP solutions exhibit acidic pH, which limits the electrochemical stability window (ESW) due to hydrogen evolution. However, the introduction of CHP effectively tunes the electrolyte pH towards near-neutral values while simultaneously enhancing ionic conductivity. The mixed CDHP + CHP electrolyte achieved improved ESW, maintaining high conductivity across a concentration range from 1 to 5 mol ⋅ kg^−1^ and down to −20°C. Electrochemical investigations on carbon/carbon symmetric capacitors using these electrolytes reveal electric double-layer (EDL) performance without redox activity in the low voltage range. However, mixed redox processes and EDL charging were observed at higher voltage, which have been evaluated with temperature-dependent electrochemical impedance spectroscopy. By modeling the Nyquist plot, absolute ion diffusion coefficients and desolvation rates have been calculated, which support the analysis of the Arrhenius plots showing that the mixed electrolyte lowers the bulk ion diffusion energy barrier for pure CDHP, driving its superior sub-zero performance.

## IMPACT STATEMENT

Choline phosphate derivatives in water produce neutral pH electrolytes due to intrinsically regulated proton activity. This is a critical design feature to expand the electrochemical stability window and low temperature performance.

## GRAPHICAL ABSTRACT

**Figure:**
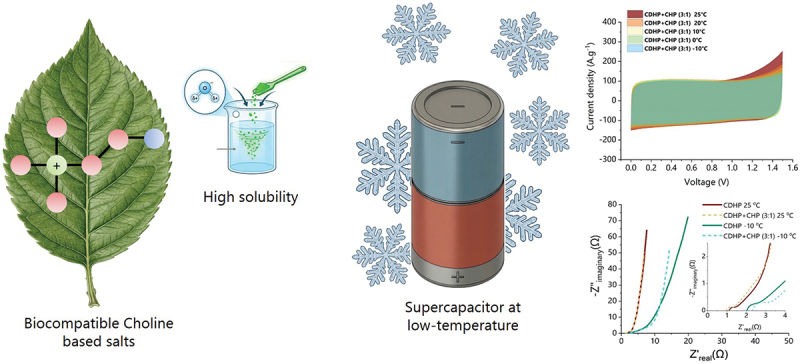

## 1. Introduction

Growing global energy demand and the shift to renewable power require reliable storage devices to manage fluctuations in energy supply. Supercapacitors (SCs), especially electric double-layer capacitors (EDLCs), store energy through a non-faradaic process at the electrode–electrolyte interface, enabling ultrafast charging and long operational lifetimes [1–3]. These characteristics make them suitable for some applications, including consumer and portable electronics, transportation, and vehicle power backup systems [4–6]. However, their broad implementation is limited by their relatively low energy density (usually below 30 Wh ⋅ kg^−1^), which is lower than batteries (around 30–200 Wh kg^−1^) [7–9]. Because the energy density scales with the square of the operating voltage [10], enhancing the cell operating voltage has become a major research focus. Importantly, the achievable operating voltage is fundamentally determined by the electrolyte, making electrolyte design a critical strategy for improving the overall energy density [11]. The commercially available electrolytes for SCs can be broadly categorized into three main types: organic electrolytes [12], ionic liquids (ILs) [13], and aqueous electrolytes [14,15]. Organic electrolytes possess a high operating voltage up to 2.7–2.85 V [16]. They are more expensive, flammable, and toxic, posing safety and environmental concerns [17–19]. ILs are promising due to their wide electrochemical stability window (ESW) (approximately 4–6 V) and good thermal stability [20–22]. On the downside, high viscosity and low electric conductivity at room temperature can reduce ion diffusion dynamics, impacting power density and rate performance [21]. Additionally, ILs are generally more expensive than other electrolytes [23]. Aqueous electrolytes offer faster charge carrier rates and improved efficiency due to their lower viscosity and higher ionic conductivity compared to non-aqueous electrolytes [24]. SCs face significant challenges in maintaining performance at low temperatures. These challenges at low temperature are electrolyte freezing [25,26], ohmic losses, sluggish ion transport [27], and capacitance reduction [27]. To address these challenges, several electrolyte engineering strategies have been proposed. High-entropy electrolytes (HEEs) [28] can suppress freezing and widen the ESW, but their high synthesis cost and complex multi-component formulations make optimization difficult and performance sensitive to slight compositional changes [29,30]. Organic electrolytes [31,32] extend the temperature range; however, the addition of organic components lowers the dielectric constant of the solvent, reduces salt solubility, promotes ionic association, and ultimately leads to low ionic conductivity [33]. Meanwhile, solid-state electrolytes (SSEs) improve operating temperature range but often suffer from low ionic conductivity and high interfacial resistance due to poor electrode compatibility [34–36]. Therefore, the development of highly conductive neutral aqueous electrolytes remains essential to maintain low assembly cost while displaying excellent low-temperature performance of supercapacitors (SCs).

Choline, naturally occurring as a quaternary ammonium cation and an essential dietary nutrient, serves as the foundation for a versatile family of highly soluble, biocompatible, and structurally tunable organic salts [37–39]. Various choline derivatives have been used to enhance the performance, safety, and operational temperature range of supercapacitors. For instance, choline bromide (ChBr) [40] has demonstrated promising potential as a novel aqueous electrolyte for hybrid supercapacitors. Also, an aqueous choline nitrate solution combined with a synthesized choline methacrylate monomer has been successfully used to prepare highly conductive hydrogel polymer electrolytes, offering a sustainable and leak-free solution for energy storage devices [41].

While various choline derivatives, have been explored as aqueous electrolytes, they fundamentally lack the intrinsic ability to regulate proton activity, leaving the system susceptible to parasitic solvent decomposition. Choline dihydrogen phosphate (CDHP) and choline hydrogen phosphate (CHP), when dissolved in distilled water, create concentrated, functionally dynamic, and neutral pH electrolytes. This near-neutral environment is a critical design feature, as it thermodynamically suppresses the hydrogen evolution reaction (HER), leverages complex internal hydrogen-bonding networks, expands the ESW, and decreases the freezing point of the electrolyte solvent.

Herein, a near-neutral aqueous choline phosphate binary electrolyte suppresses HER, expanding the supercapacitor voltage window to 1.5 V and enabling superior anti-freezing charge storage down to −15°C. The electrochemical performance of proposed electrolytes was evaluated in a symmetric supercapacitor cell across a wide temperature range using cyclic voltammetry (CV), galvanostatic charge–discharge (GCD), and electrochemical impedance spectroscopy (EIS). Through these analyses, the operating voltage windows (up to 1.5 V), specific discharge capacitance, and the resulting specific energy and power were mapped, while resistance parameters were extracted using EIS to quantify the activation energy barriers governing ion diffusion and desolvation.

## 2. Experimental methods: electrolyte preparation, characterization, and supercapacitor cell assembly

### 2.1. Materials

Choline chloride (ChCl ≥ 98%) and ethanol (EtOH, 96%) was supplied from Sigma Aldrich (St. Louis, MO, U.S.A.). Sodium hydroxide (NaOH, 98.8%) and orthophosphoric acid (H_3_PO_4_, 85 wt.% in H_2_O) were provided by Chempur (Piekary Śląskie, Poland).

### 2.2. Synthesis of choline dihydrogen phosphate (CDHP) and choline hydrogen phosphate (CHP)

Choline dihydrogen phosphate is commonly synthesized via ion-exchange methods [42] or in electrodialysis metathesis [43], which often require additional purification steps to remove inorganic by-products or involve the use of specialized materials such as ion-exchange resins. While these methods provide high-purity products, they may be time-consuming or less convenient for systematic studies. In this work, we used a new approach based on the *in situ* generation of choline hydroxide followed by controlled neutralization with phosphoric acid. It allows straightforward control of the reaction stoichiometry. As a result, not only CDHP but also CHP can be obtained.

**Figure 1.**
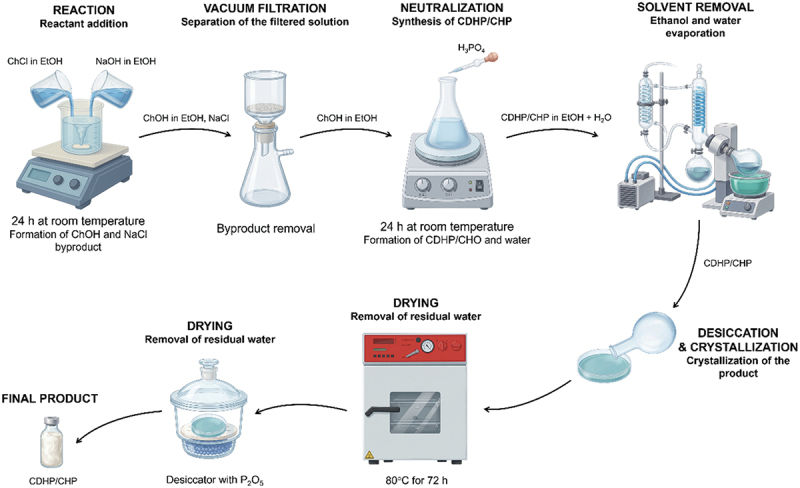
Schematic representation for synthesizing choline-based salts. The process illustrates the *in situ* generation of choline hydroxide from choline chloride and sodium hydroxide in ethanol, followed by byproduct removal via vacuum filtration. Subsequent neutralization with phosphoric acid produces the target salts, which are isolated through solvent evaporation, thermal drying at 80°C, and final desiccation with P_2_O_5_.

Sodium hydroxide (1 mol) and choline chloride (1 mol) were dissolved in ethanol (1 L), then mixed in an equimolar ratio of NaOH to ChCl and stirred on a magnetic stirrer at room temperature for 24 h. The reaction proceeded according to Equation (1). Subsequently, the NaCl residue was filtered, obtaining pure choline hydroxide in an ethanol solution. The solution was titrated to determine the exact concentration. Next, the solution was mixed with a proper molar ratio of phosphoric acid (1:1 to obtain CDHP and 2:1 to obtain CHP) and stirred on a magnetic stirrer at room temperature for 24 h. The reactions proceeded according to Equation (2) and Equation (3). The solvent was evaporated from the liquid phase using a rotary evaporator. The CDHP and CHP salts were placed in a drying chamber VD 23 (BINDER, Tuttlingen, Germany) for 72 h at 80°C and then in a desiccator containing P_2_O_5_ drying agent to remove excess water. The details of the synthesis method are schematically shown in Figure 1.

**Figure:**
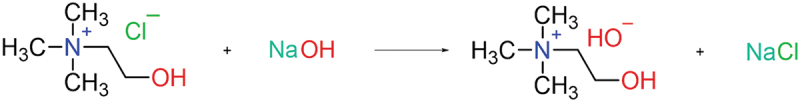

**Figure:**
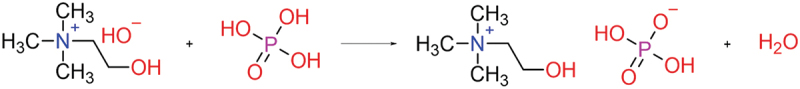

**Figure:**
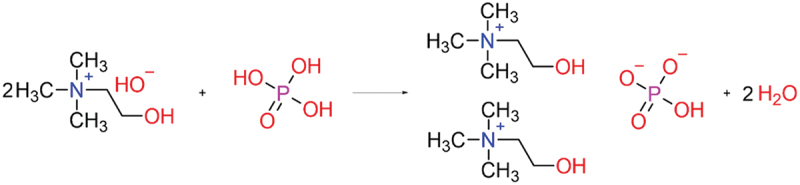

### 2.3. Preparation of electrolyte

CDHP electrolytes were prepared by dissolving a proper amount of salt in distilled water to obtain molalities of 1, 2, 3, 4, and 5 mol⋅kg^−1^. Mixed electrolytes were obtained by dissolving appropriate amounts of CDHP and CHP at a molar ratio of 3:1 in distilled water to obtain molalities of 1, 2, 3, 4, and 5 mol⋅kg^−1^.

### 2.4. Electrode preparation

The electrodes were fabricated from a slurry containing 90 wt.% activated carbon (Maxsorb MSP-20X, Kansai Coke and Chemicals Co., Hyogo, Japan) with 5 wt.% carbon black (C65, Imerys) and 5 wt.% polytetrafluoroethylene (PTFE). Maxsorb MSP-20 is a microporous carbon characterized by a highly developed porous structure, featuring a mean pore size of 0.81 nm. Furthermore, it possesses a total pore volume of 0.84 cm^3^⋅ g^−1^ and a specific surface area of 1763 m^2^⋅ g^−1^ [44]. N,N-dimethylformamide (DMF) was used as the dispersion medium electrode components. The mixture was homogenized thoroughly and then rolled out into thin films. After solvent evaporation at room temperature, the films were dried at 70°C for 12 h to remove the residual DMF. Subsequently, the films were carefully detached from the substrate, and circular electrodes with a diameter of 12 mm and a thickness of 175 μm were punched out, which then were attached to 316 L stainless-steel current collectors using Electrodag PF-407C conductive adhesive (Henkel, Düsseldorf, Germany).

### 2.5. Cell assembly

Symmetric supercapacitor cells were assembled in a two-electrode Swagelok®-type cell configuration, using 316 L stainless steel current collectors. Prior to assembly, the electrodes were impregnated with the electrolyte, and a glass fiber separator (Whatman GF/A, 260 µm thick) was positioned between the electrodes.

### 2.6. Physicochemical characterization

Fourier transform infrared (FTIR) spectra were recorded using a Nicolet 5700 spectrometer equipped with an attenuated total reflectance (ATR) accessory fitted with a diamond crystal. Measurements were performed in the wavenumber range of 4000–525 cm^−1^ at a spectral resolution of 4 cm^−1^, using 32 scans per spectrum, at room temperature. The analyses were conducted for pure salts, their aqueous solutions, and their mixture.

The pH of electrolytes was measured by pH-meter (Elmetron) at room temperature. The viscosity was measured by a cone-plate viscometer at 25°C (Brookfield RVDV-II+ Pro). Ionic conductivity of electrolytes was measured at room temperature using an electrochemical cell dedicated to liquid samples and calculated according to Equation 4.

(4) $$\sigma =k\cdot \sigma_{s}$$

where *σ* is the ionic conductivity (mS·cm^−1^), *k* is the cell constant, equal to 1.54 cm^−1^, and *σ*_*s*_ corresponds to the measured conductivity of tested electrolyte (mS).

Conductivity measurements were carried out in a two-electrode Swagelok®-type cell, where electrolyte was placed in a Teflon cylinder, positioned in-between metallic current collectors. Temperature-dependent conductivity measurements were additionally performed using a BINDER MK56 climatic chamber within the temperature range from 25°C to −40°C with 5°C intervals after 1.5 h stabilization period at each temperature.

To evaluate the phase transition behaviors of the electrolytes at sub-zero temperatures, thermal analysis was performed. The crystallization and melting temperatures of the 2 mol ⋅ kg^−1^ CDHP and 3 mol ⋅ kg^−1^ CDHP + CHP (3:1) electrolytes were determined using differential scanning calorimetry (DSC) using a DSC 204 F1 Phoenix® apparatus from Netzsch (Selb, Germany). The electrolyte samples were placed in an aluminum crucibles and then closed with a lid. Subsequently, samples were analyzed within the temperature range 25°C to −60°C, under a nitrogen atmosphere. Heating and cooling cycles were performed at a rate of 5°C·min^−1^.

### 2.7. Electrochemical tests

Electrochemical performance was conducted using symmetric two-electrode Swagelok®-type supercapacitor cells connected to an SP-300 potentiostat/galvanostat (Biologic, Seyssinet-Pariset, France). CV measurements were performed within a potential window of 0.8–1.5 V at 2 mV·s^−1^ scan rate. GCD measurements were carried out at current densities between 0.2 and 4 A·g^−1^, calculated based on the average mass of activated carbon in a single electrode. The GCD curves were used to calculate energy efficiency (%) based on Equation (5) [45]:

(5) $$\eta_{E}=\frac{E_{\mathrm{int}/D}}{E_{\mathrm{int}/C}}\cdot 100\%,$$

where *E*_*int/D*_ and *E*_*int/C*_ (V⋅s) correspond to the areas under the galvanostatic discharge and charge curves, respectively. Additionally, the average electrode-specific capacitance (F·g^−1^) was calculated according to Equation (6) [45]:

(6) $$C_{\mathrm{el}.\mathrm{int}/D}=\frac{2\cdot I\cdot E_{\mathrm{int}/D}}{0.5\cdot m_{\mathrm{el}}\cdot \Delta U^{2}},$$

where *I* is the applied current (A), *m*_*el*_ (g) is the mass of an electrode, and *ΔU* is the change in cell potential (V).

EIS was performed at open-circuit potential over a frequency range from 1 MHz to 1 mHz with a sinusoidal perturbation amplitude of 7 mV.

## 3. Result and discussion

### 3.1. Physicochemical properties of electrolytes

**Figure 2.**
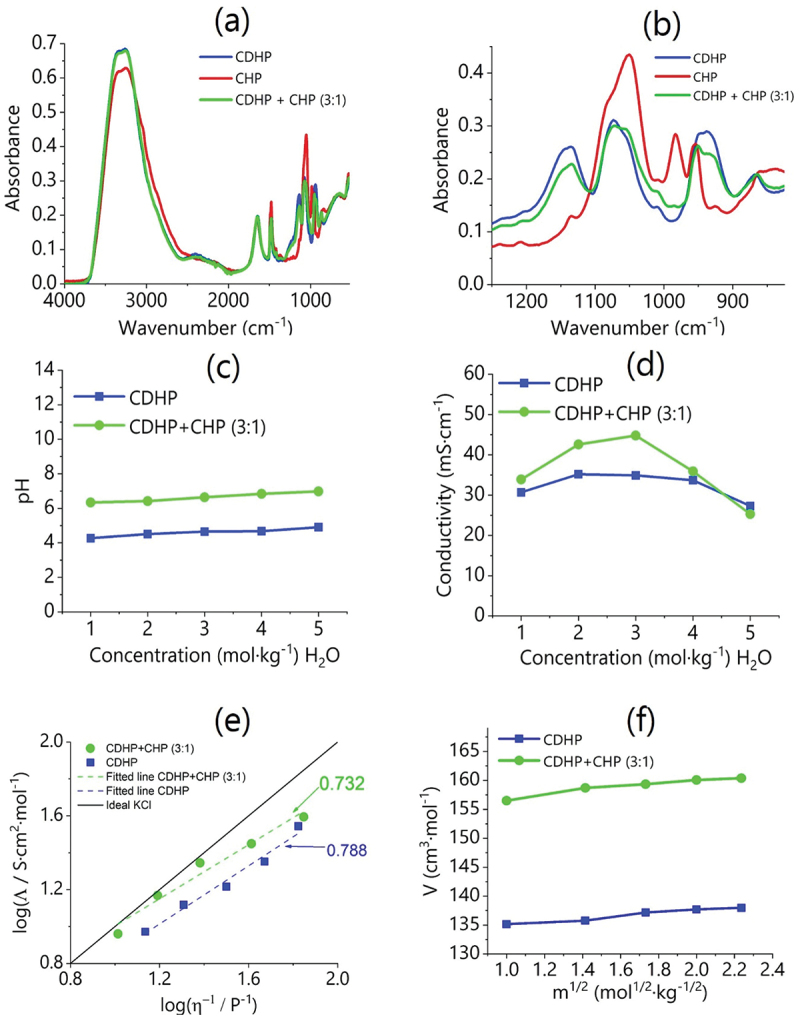
FTIR – ATR spectra of aqueous CDHP and CHP solutions and their mixture (CDHP+CHP = 3:1) at 5 mol kg^−1^ H_2_O: (a) Full spectral range and (b) Region characteristic of phosphate vibrations. Ionic conducitivty, pH and transport kinetics of pure CDHP and mixed CDHP + CHP (3:1) aqueous electrolytes at 25°C. The panels illustrate: (c) Concentration-dependent pH profiles demonstrating the near-neutral shift in the mixed system; (d) Ionic conductivity as a function of salt concentration ; (e) Fractional Walden plot assessing ion transport behavior and the decoupling of ions from bulk viscosity and (f) The dependence of apparent molar volume on the square root of molality (m^0.5^), modeled according to Redlich-Rosenfeld-Meyer’s equation to evaluate solute-solute and solute-solvent interactions.

The ATR-FTIR spectra of the CDHP and CHP salts and electrolytes are presented in Figure 2 and S1. The characteristic absorption bands corresponding to the principal functional groups are indicated, including the hydroxyl groups associated with both the choline cation and the phosphate anions, as well as the phosphate moieties of the anions [46]. The 3600–2800 cm^−1^ region is characterized by a broad composite absorption band originating from overlapping O–H and C–H stretching vibrations. The broad O–H stretching component, associated with hydroxyl groups engaged in hydrogen bonding, is superimposed on the alkyl stretching bands of the choline cation. In addition, the bands observed between 1500 and 1460 cm^−1^ can be assigned to CH_2_ bending vibrations of the choline cation [47]. Furthermore, the characteristic C–N stretching vibration of the quaternary ammonium group appears at 957 cm^−1^ for CDHP and 953 cm^−1^ for CHP. The absorption bands observed in the 1150–950 cm^−1^ region are characteristic of phosphate-containing species [48–52]. For the CDHP salt, the phosphate-related bands were assigned to the asymmetric stretching vibration of the P–OH bond at 924 cm^−1^, the symmetric stretching vibration of the P–O bond at 1076 cm^−1^, and the asymmetric stretching vibration of the P–O bond at 1158 cm^−1^. In the CHP spectrum, a weak band at 926 cm^−1^ was attributed to the asymmetric stretching vibration of the P–OH bond, whereas the bands at 977 and 1032 cm^−1^ correspond to the symmetric and asymmetric stretching vibrations of the P–O bond, respectively. The substantially lower intensity of the P–OH band in the CHP spectrum is consistent with the lower abundance of P–OH groups compared with CDHP.

In aqueous solution, the absorption bands of the investigated salts become noticeably broadened and exhibit shifts in their positions, reflecting changes in the local chemical environment and intermolecular interactions resulting from solvation. As shown in Figure 2a-b, the spectra of the aqueous CDHP and CHP solutions, as well as their mixed solution (CDHP+CHP), are dominated in the 3700–2600 cm^−1^ region by a broad absorption band arising from water molecules and strongly hydrogen-bonded O–H groups. In addition, a sharp band of medium intensity with a maximum at 1644 cm^−1^, assigned to the H–O–H bending vibration of water molecules, is observed [53]. The phosphate-related bands of the investigated salts remain detectable in the 1200–900 cm^−1^ region, although they are considerably broadened. Notably, the absorption band observed at 1158 cm^−1^ in the spectrum of solid CDHP shifts toward lower wavenumbers upon dissolution and appears as a shoulder superimposed on the band associated with the choline cation. A similar feature is observed in the spectrum of the mixed-salt solution (CDHP+CHP), indicating that specific intermolecular interactions are retained in the mixed electrolyte system. The band at 1076 cm^−1^, assigned to the symmetric stretching vibration of the P–O bond, is clearly visible in both the aqueous CDHP solution and the mixed-salt solution. Furthermore, the mixed-salt solution exhibits a band characteristic of CHP (approximately 1030 cm^−1^), assigned to the asymmetric stretching vibration of the P–O bond. Upon dissolution in water, this band shifts to a higher wavenumber (1050 cm^−1^) and appears at the same position in the spectrum of the CDHP+CHP solution. Similarly, the band corresponding to the symmetric stretching vibration of the P–O bond in CHP shifts to 983 cm^−1^ in aqueous solution. This band is present in both the CHP solution and the mixed-salt solution, although its intensity is relatively low in the latter case. Moreover, an absorption band located at approximately 925 cm^−1^ is observed in all investigated salt solutions, suggesting that this vibration is associated with structural motifs common to both phosphate-containing salts. Weak bands detected in all aqueous solutions within the 885–850 cm^−1^ region are attributed predominantly to P–O(H) vibrations related to hydrogen phosphate species [48]. Overall, the ATR – FTIR analysis confirms that the chemical structures of CDHP and CHP remain unchanged in aqueous solution and in the mixed electrolyte system. Although no new absorption bands indicative of chemical reactions were detected, the observed shifts and broadening of characteristic bands suggest the presence of specific intermolecular interactions, including solvation effects and hydrogen-related interactions involving both the phosphate-containing anions and water molecules.

The viscosity of both aqueous electrolytes has been measured at room temperature from 1 to 5 molal concentration of the salt (Figure S2a). The viscosity increases with increasing concentration for both electrolytes, which is typical for electrolyte solutions due to enhanced intermolecular interactions and reduced molecular mobility. The CDHP + CHP (3:1) mixture exhibits slightly higher viscosity values compared to CDHP, particularly at higher concentrations. This behaviour suggests stronger interaction between ionic species in the mixed electrolyte, likely associated with increased ion-ion interactions and possible formation of transient aggregates.

The DSC analysis (Figure S2b) reveals differences between the 2 mol·kg^−1^ CDHP and 3 mol·kg^−1^ CDHP + CHP (3:1) electrolytes. For CDHP, a sharp exothermic peak at −34.8°C is observed, indicating a crystallization process. The high intensity and narrow shape of this peak suggest rapid formation of well-defined crystalline phase. On the contrary, the CDHP + CHP (3:1) exhibits a significantly weaker and broader exothermic signal shifted to −50.9°C. This behaviour indicates that crystallization is less pronounced. In addition, weak endothermic transitions are observed at −8.4°C for CDHP and at −20.9°C for CDHP + CHP (3:1). Their low intensity and broad character suggest that they are associated with thermal transformations within the solid phase, such as melting or structural reorganization.

Figure 2c illustrates the concentration-dependent pH of the prepared aqueous electrolytes. Pure CDHP solutions exhibit a distinctly acidic pH across the measured concentration range, which is not desirable for SCs [54]. However, the strategic introduction of the dibasic CHP salt effectively tunes the mixed electrolyte (CDHP + CHP) towards near-neutral pH values. As shown in this figure, the binary mixture maintains this stable, near-neutral pH consistently from 1 to 5 molal concentrations. This neutralization is a critical design feature, as it mitigates parasitic hydrogen evolution reaction (HER) activity and ultimately extends the stable operating voltage limits of the electrolyte.

The conductivity of both electrolytes was investigated at different concentrations at room temperature (Figure 2d). The conductivity initially increases with concentration for both systems, reaching a maximum at intermediate concentration, 2 mol⋅kg^−1^ H_2_O for CDHP and at 3 mol⋅kg^−1^ H_2_O for CDHP + CHP (3:1), and then decreasing at higher concentrations. This behaviour reflects the balance between the increasing number of charge carriers and the simultaneous increase in viscosity, which limits ionic mobility [55]. The CDHP + CHP (3:1) system exhibits higher conductivity values compared to CDHP over most of the concentration range, indicating a higher effective number of charge carriers and/or enhanced ion mobility. This may be attributed to the presence of multiply charged phosphate species and reduced ion-solvent interactions. Also, the conductivity data for these two electrolytes in the temperature range of 25°C to −40°C can be found in the Supporting Information. Figure S3 shows the temperature dependence of ionic conductivity for CDHP and CDHP + CHP (3:1) electrolytes at concentrations ranging from 1 to 5 mol ⋅ kg^−1^ H_2_O. In both cases, ionic conductivity increases with temperature due to enhanced ionic mobility. Its dependence on concentration reveals an optimum at 2 and 3 mol ⋅ kg^−1^ H_2_O, where conductivity is highest. Compared to pure CDHP, the CDHP + CHP mixture exhibits overall higher conductivity, especially at moderate concentrations. Notably, in both cases, a distinct jump in conductivity is observed around −15°C to −10°C, which may indicate a structural transition in the system. Overall, the results indicate that the addition of CHP modifies the solution properties by increasing pH, enhancing viscosity, and improving conductivity at moderate concentrations, highlighting the significant role of phosphate speciation in determining the physicochemical behaviour of the system.

To evaluate the ion transport kinetics within the aqueous choline phosphate systems, the relationships between physical measurements (viscosity and conductivity) were analyzed for both electrolytes, as depicted in Figure 2e. The transport behavior of the electrolytes was initially assessed at 25°C using the empirical fractional Walden rule, which correlates molar conductivity (Λ) with fluidity (η^−1^). The molar conductivity was derived from the measured ionic conductivity (σ) and concentration (c) via Equation 7 [56].

(7) $$\Lambda =\frac{\sigma }{c}$$

The fractional Walden behavior is expressed logarithmically as Equation 8:

(8) $$\log\left(A\right)=\log\left(C\right)+\alpha \log\left(\eta^{-1}\right)$$

A linear dependence was observed for both investigated solutions, confirming that ion transport within these specific choline phosphate electrolytes adheres to fractional Walden behavior. The extracted slope values (*α*) for both systems were strictly less than 1, yielding *α* = 0.788 for the CDHP electrolyte and a lower *α* = 0.732 for the mixed CDHP + CHP system. A fractional parameter of *α* < 1 indicates that the ions are largely decoupled from the macroscopic viscosity of the bulk fluid, moving faster than the bulk viscosity would theoretically suggest. This deviation implies that ion transport is not solely governed by hydrodynamics but is heavily influenced by intricate ion-ion and ion-solvent interactions. The depressed *α* value for the mixed system suggests that the addition of CHP further strengthens these intermolecular interactions within the solvent.

To quantify the energy barriers limiting ion migration at sub-zero temperatures, the temperature-dependent ionic conductivity was evaluated from 25°C down to −40°C. As the external temperature decreases, the thermal energy available for ions to jump between coordination sites diminishes, and the solvent viscosity exponentially increases. The temperature dependence of ionic conductivity was modeled using the Arrhenius relation (Equation 9):

(9) $$\sigma =\mathrm{Aexp}\left(-\frac{E_{a}}{\mathrm{RT}}\right)$$

Taking the natural logarithm yields a linear equation (Equation 10) to extract the activation energy (*E*_*a*_), where *R* is the universal gas constant, and *A* is the pre-exponential factor:

(10) $$\mathrm{Ln}(\sigma )=\ln\left(A\right)-\left(\frac{E_{a}}{\mathrm{RT}}\right)\cdot \left(\frac{1}{T}\right)$$

The calculated values of activation energy increase with electrolyte concentration, ranging from ~19 kJ ⋅ mol^−1^ at lower concentrations to about 26 kJ.mol^−1^. That trend indicates that ion transport becomes increasingly hindered as the electrolyte concentration increases. At comparable concentrations, similar activation energy values were observed for both CDHP and CDHP + CHP (3:1) electrolytes, suggesting a comparable mechanism of ionic transport in both systems.

In order to further examine the effect of electrolyte composition on intermolecular interaction, the apparent molar volumes *V*_*ϕ*_ (cm^3^⋅mol^−1^) were calculated by Equation 11 [57,58]:

(11) $$V_{\phi }=\frac{M}{d}-\frac{1000\cdot \left(d-d_{0}\right)}{m\cdot d\cdot d_{0}}$$

Where *M* is the molar mass of the salt (g ⋅ mol^−1^) *d* and *d*_*0*_ (g ⋅ cm^3^) are the density of the solution and the pure solvent, respectively, and *m* denotes the number of moles of the salts per kilogram of pure solvent (mol ⋅ kg^−1^). In the case of a mixture of salts, the molar mass *M* was calculated as a weighted average of the molar masses of individual components, using their respective molar fractions.

The apparent molar volume data were treated according to the Redlich-Rosenfeld-Meyer’s equation [57,58]:

(12) $$V_{\phi }=V_{\phi }^{0}+S_{v}m^{1/2}+B_{v}m$$

Linear plots of *V*_*ϕ*_ versus *m*^*1/2*^ were constructed (Figure 2f), and the experimental slope was used to determine the limiting apparent molar volume *V*_*ϕ*_^*0*^ (cm^3^⋅ mol^−1^), which is the intercept of the linear fit and describes the value of the *V*_*ϕ*_ at infinite dilution and the slope corresponds to the *S*_*v*_ (cm^3^⋅ mol^−3/2^⋅ kg^1/2^) parameter, providing information on solute-solute interactions and *B*_*v*_ (cm^3^⋅ mol^−2^) is fitted empirical parameter. Based on the linear fits of the experimental data using Redlich-Rosenfeld-Meyer’s equation, the extracted empirical parameters for both the CDHP and mixed CDHP + CHP (3:1) electrolytes are presented in Table 1.

**Table 1.** The apparent molar volume at infinite dilution *V*^0^_*ϕ*_, S_v_, B_v_, parameters calculated using Eq. (12) for CDHP and CDHP + CHP mixture in water at temperature 25°C.

Electrolyte	*V*^0^_*ϕ*_, cm^3^·mol^−1^	*S_v_*, cm^3^·mol^−3/2^·kg^1/2^	*B_v_*, cm^3^·mol^−2^
CDHP	132.2	3.0	-0.16
CDHP + CHP	149.0	9.5	-1.99

The limiting apparent molar volume *V*_*ϕ*_^*0*^ refers to the partial molar volume of a solute at infinite dilution, where solute-solvent interactions dominate, and solute-solute interactions are negligible. It arises from several contributions, including the intrinsic ionic volume, volume changes from electrostriction, and structural volume changing from the surrounding solvent. As reported in the literature [57,59,60], *V*_*ϕ*_^*0*^, in electrolyte and ionic liquid systems is governed not only by ion-solvent interactions but also by ion size and solution structuring effects.

In the present study, the *V*_*ϕ*_^*0*^, value for CDHP (132.2 cm^3^⋅ mol^−1^) is lower than that for the CDHP + CHP (3:1) system (149.0 cm^3^⋅ mol^−1^). The lower *V*_*ϕ*_^*0*^ for CDHP indicates a greater contraction of the solvent structure, which may be attributed to a larger contribution of electrostrictive effects associated with stronger ion-solvent interactions. In contrast, the higher *V*_*ϕ*_^*0*^ value observed for the CDHP + CHP (3:1) suggests a reduction in volumetric contraction and a greater contribution of intrinsic ionic volume, as well as possible structural reorganization of the solution. Similar behaviour has been reported for ionic liquid systems, where increases in *V*_*ϕ*_^*0*^ are associated with changes in ionic structure and enhanced contribution of intrinsic ionic volume rather than a simple weakening of interactions [60].

The positive values of *S*_*v*_ obtained for both electrolytes indicate the presence of significant ion-ion interactions, while the higher *S*_*v*_ value for CDHP + CHP (3:1) (9.5 cm^3^⋅ mol^−3/2^⋅ kg^1/2^) compared to CDHP (3.0183 cm^3^⋅ mol^−3/2^⋅ kg^1/2^) suggests enhanced ion association in the mixed electrolyte [3]. The negative values of *B*_*v*_ indicate the presence of a solvent-induced solute co-sphere overlap effect, suggesting enhanced interactions between ionic species, accompanied by partial dehydration and the release of water molecules into the bulk phase [58].

The simultaneous increase in *V*_*ϕ*_^*0*^ and *S*_*v*_ upon the addition of CHP indicates a change in the balance of interactions in the system. The higher *V*_*ϕ*_^*0*^ suggests a reduced contribution of volumetric contraction effects, while the increase in *S*_*v*_ points to enhanced interaction effects between solute species. However, these parameters should be interpreted as empirical indicators, and no definitive conclusions about the dominant interaction mechanism can be drawn based solely on volumetric data.

### 3.2. Hydrogen evolution and two-electrode cell performance comparison

**Figure 3.**
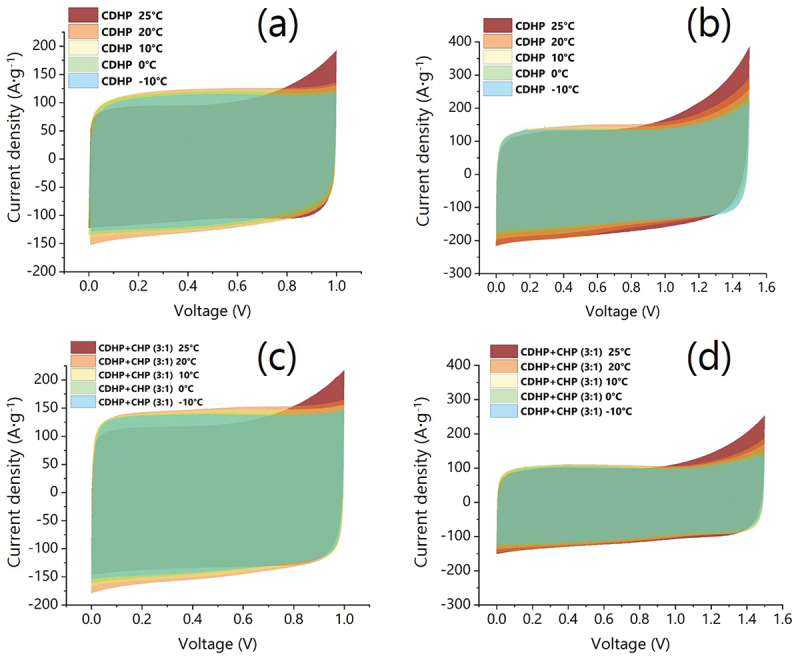
Cyclic voltammetry plots for CDHP at: (a) 1.0 V and (b) 1.5 V and for 2CDHP+CHP (3:1) (c) 1.0 V and (d) 1.5 V. The scan rate for all CVs is 2 mV s^−1^.

The CV profiles illustrated in Figure 3 evaluate the charge storage behavior and the operational voltage limits of the assembled symmetric supercapacitors. Figures 3a-b display the CV curves for the pure choline dihydrogen phosphate (CDHP) electrolyte operated at maximum cell voltages of 1.0 V and 1.5 V, respectively. The surface area of CV for CDHP is higher, which corresponds to higher capacitance [61]. According to these plots, as the temperature decreases, the shape of the CV plot evolves to a rectangular shape, implying an ideal EDL. This change in the charge-storage mechanism is due to the suppression of electrolyte decomposition reactions at lower temperatures [62].

Figures 3c-d present the corresponding CV plots for the mixed CDHP + CHP (3:1) electrolyte at 1.0 V and 1.5 V. Across all evaluated conditions, the CV curves demonstrate a quasi-rectangular shape indicative of classic EDLC. Furthermore, the absence of any distinct faradaic peaks indicates excellent electrochemical stability without any redox activity. In comparison with CDHP, the mixed electrolyte possesses less surface area in both 1.0 and 1.5 V voltage windows. Importantly, in comparison with the CDHP electrolyte, the mixed electrolyte has a less sharp increase. This expanded operating voltage window is critical, as it translates directly into a higher overall energy density for the electrochemical capacitor. Moreover, with increasing temperature, the value of surface area of CV curves increases. According to the physicochemical data of electrolytes, higher temperatures generally enhance ionic conductivity and consequently ion adsorption/desorption, leading to increased specific capacitance.

To further elucidate the charge storage mechanism and quantify the temperature-dependent capacitive response, the accumulated specific charge was evaluated as a function of the applied working potential at both 25°C and −10°C in Figure S4a. The resulting charge–potential profiles exhibit a highly linear dependence across the entire 1.5 V operational window for all tested conditions. This pronounced linearity is a fundamental characteristic of ideal electric double-layer behavior, confirming a constant state of capacitance and the absence of parasitic faradaic reactions within the extended stability window. Furthermore, the narrow hysteresis observed between the anodic and cathodic sweeps across both temperatures signifies excellent electrochemical reversibility. At ambient temperature (25 °C), the pure CDHP electrolyte possesses a distinctly steeper slope than the CDHP+CHP (3:1) electrolyte. Because the slope directly corresponds to the specific capacitance, this visually demonstrates that the pure CDHP electrolyte facilitates greater specific charge accumulation. This corroborates the larger integrated surface area observed in the standard cyclic voltammetry profiles for the pure electrolyte. Upon lowering the operational temperature to −10°C, a reduction in the slope is observed for both electrolyte systems. This indicates a predictable decrease in specific capacitance, which occurs because the thermal energy available for ion migration diminishes and the viscosity of the aqueous solvent increases at lower temperatures. While the pure CDHP electrolyte retains a higher absolute charge accumulation at −10°C, both systems demonstrate robust low-temperature functionality. Notably, even at low operating temperatures, the electrolytes maintain their highly linear, purely capacitive charge-storage mechanisms without significant resistive deformation.

In order to investigate the dynamic charging kinetics, high-voltage stability, and charge storage efficiency, the cyclic voltammetry data was translated into a time-dependent current density profile (Figure S4b). While an ideal electric double-layer capacitor exhibits perfectly horizontal current plateaus, real systems often deviate at extreme potentials. During the initial sweep (e.g. 25–200 s), both electrolytes demonstrate relatively flat, purely capacitive responses. However, as the potential approaches the 1.5 V upper limit (near 300 s), the pure CDHP electrolyte exhibits a sharp, exponential current surge. This upward drift signifies the onset of severe leakage current and parasitic hydrogen evolution reaction (HER) driven by the acidic nature of the pure solution. Consequently, while integrating the area under the I vs. t curve (Q = $\int^{I}\cdot \mathrm{dt}$) for pure CDHP yields a large total accumulated charge, a significant fraction of this area is artificially inflated by irreversible solvent decomposition. In stark contrast, the mixed CDHP + CHP (3:1) electrolyte successfully mitigates this HER activity, maintaining a highly stabilized current plateau at the voltage vertex. Therefore, although the absolute integrated charge is slightly lower than that of the pure electrolyte, the area under the mixed electrolyte’s curve corresponds almost exclusively to highly efficient, reversible electrostatic storage. By suppressing parasitic faradaic reactions, the binary mixture ensures that electrical energy is stored purely capacitively, validating its superior electrochemical stability and enhanced coulombic efficiency at expanded cell voltages.

**Figure 4.**
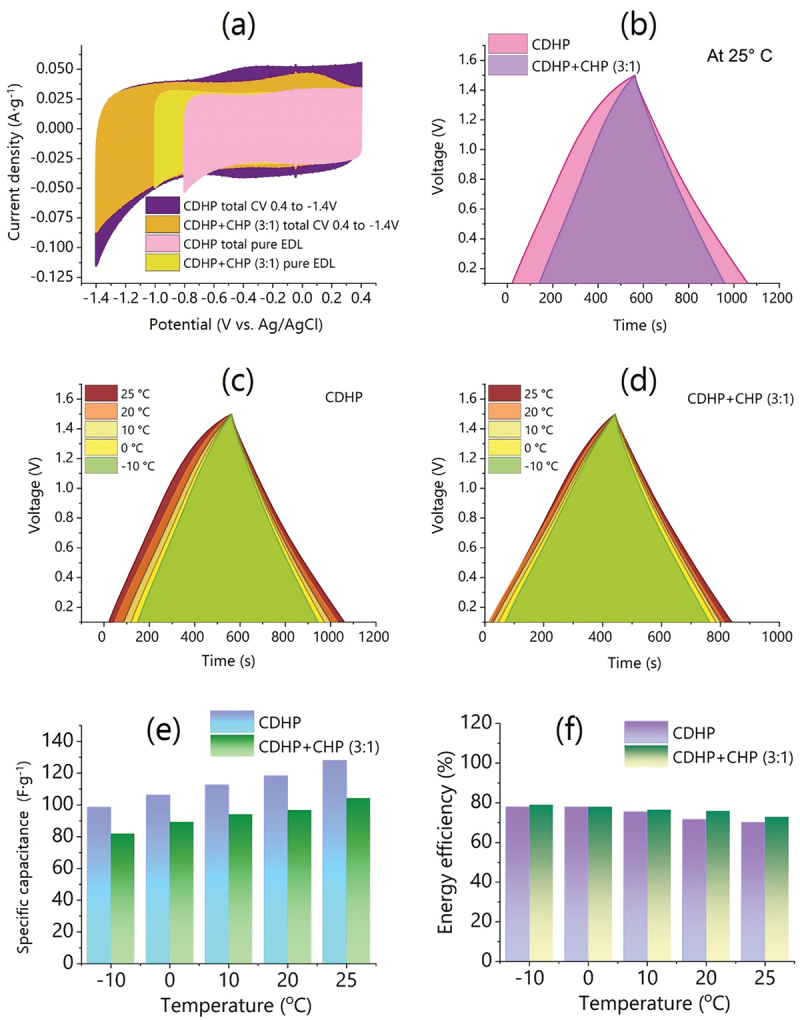
(a) Comparing the HER activity and the share of pure EDL in two electrolytes at room temperature. (b) Comparison of the GCD performance of two electrolytes at 25 °C. (c and d) GCD plots at different temperatures for the electrolytes. The values of (e) Specific capacitance and (f) Energy efficiency at various temperatures.

Figure 4 provides a comprehensive analysis of the aqueous electrolyte reduction process at negative potentials, specifically the hydrogen evolution reaction (HER), alongside their temperature-dependent charge-discharge efficiencies. Figure S5a and b monitor the HER activity in negative voltage regions, specifically comparing the two electrolytes in the 0.4 V to −1.4 V range. Pure CDHP solutions exhibit an acidic pH, which fundamentally limits the ESW by promoting early hydrogen evolution. However, introducing the dibasic CHP salt effectively tunes the electrolyte towards near-neutral pH values.

According to the Nernst equation (detailed in method S1), this pH neutralization thermodynamically shifts the theoretical reduction potential for HER from −0.478 V down to −0.604 V (vs. Ag/AgCl), actively suppressing water decomposition. As observed in the comparative analysis in Figure 4a, the share of pure EDL charge storage was evaluated. Specifically, within the voltage limits of −0.8 V for pure CDHP and −1.0 V for the CDHP+CHP (3:1) mixture, the integrated areas under the charging and discharging curves (using a standard Y = 0 baseline in Figure S5c) are virtually identical. This symmetry confirms ideal, pure EDL behavior in these regimes. However, by calculating the absolute integrated area of the scan at the −1.4 V limit, it becomes evident that the pure CDHP electrolyte exhibits a substantially higher total current consumption. This inflated area does not represent stored electrostatic energy; rather, it serves as a direct indicator of severe parasitic electrolyte decomposition reactions. Ultimately, the binary mixture successfully suppresses these non-capacitive losses while physically storing more genuine electrostatic charge.

Figure 4b directly compares the GCD performance of both pure and mixed electrolytes at 25°C. According to this comparison, the mixed electrolyte shows shorter charging and discharging times, but exhibits a shape closer to the ideal triangle characteristic of EDL formation. Figures 4c,d expand this analysis by illustrating the GCD profiles across a broad temperature range (from 25°C down to −10 °C). At 25°C, CDHP and mixed electrolyte achieved a specific capacitance of 128.1 and 104.3 F ⋅ g^−1^ and an energy efficiency of 70.2% and 73.0%, respectively. When the temperature was lowered to −10°C, the CDHP + CHP (3:1) mixture maintained a specific capacitance of 82 F ⋅ g^−1^ and an energy efficiency of 79% compared to 99 F ⋅ g^−1^ and 78% for CDHP.

The highly linear and symmetrical nature of these charge-discharge curves confirms the excellent reversibility and capacitive response of the porous carbon electrodes within these aqueous systems. Importantly, with decreasing temperature, the GCD plot undergoes horizontal compression because of the limitation of ion transport. The bar plots extract and summarize the specific capacitance (Figure 4e) and energy efficiency (Figure 4f) at various temperatures. The galvanostatic measurements reveal enhanced specific capacitance for the mixed electrolyte setup. Notably, the CDHP + CHP mixture maintains superior specific capacitance and ionic conductivity at subzero temperatures.

To evaluate the single-electrode performance and validate the optimized 1.5 V operating window, the specific discharge capacitance of the individual positive and negative electrodes was analyzed using a three-electrode configuration (Figure S6). As shown in the capacitance versus potential range profiles, the negative electrode consistently exhibits a higher specific capacitance than the positive electrode across all evaluated voltages for both the pure CDHP and mixed CDHP + CHP (3:1) electrolytes. Most importantly, the pure CDHP system exhibits a sharp drop in specific capacitance beyond 1.8 V, indicating severe instability and electrolyte decomposition. In contrast, the capacitance values for the mixed CDHP + CHP system remain relatively stable and linear at elevated potentials, directly confirming that the buffering effect of the binary mixture effectively stabilizes the cell for optimized operation at 1.5 V.

The specific energy density (E) of the cell is directly proportional to its specific capacitance, according to Equation 13 [63]:

(13) $$E=\frac{1}{2}CV^{2}$$

Using this equation, the relationship between energy density and temperature has been plotted in Figure S7. The slightly lower slope obtained for the mixed electrolyte suggests a marginally weaker dependence of specific energy on temperature, indicating somewhat improved energy retention during cooling. Nevertheless, a statistical comparison of the regression slopes (α = 0.05) showed that the observed difference was not significant. Therefore, despite small quantitative differences between the systems, the rate of specific energy loss with decreasing temperature can be considered comparable for both electrolytes. Within the limits of experimental uncertainty, the temperature dependence of the specific energy is thus essentially similar for pure CDHP and mixed CDHP+CHP electrolyte systems. Overall, the analysis of the capacitance retention as a function of temperature demonstrates that the supercapacitor maintains satisfactory electrochemical performance down to −10°C for CDHP and to −15°C for mixed electrolyte (Figure S8). The analysis of the capacitance retention as a function of temperature demonstrates that the supercapacitors maintain satisfactory electrochemical performance down to −10°C for CDHP and to −15°C for mixed CDHP+CHP electrolyte.

### 3.3. Impedance response of electrolytes in porous carbon electrodes at different temperatures

**Figure 5.**
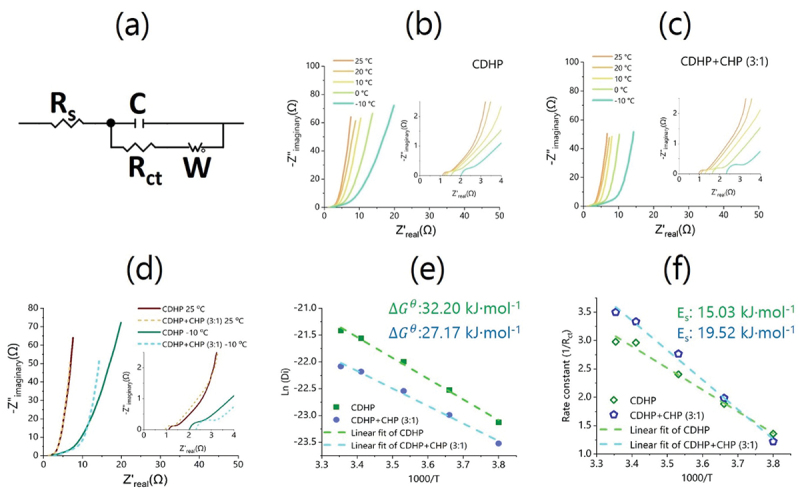
(a) Equivalent circuit model used for simulating EIS data. Nyquist plot for (b) CDHP and (c) CDHP+CHP electrolytes at different temperatures. (d) Changes of Nyquist plot with varying the temperature. Arrhenius plots for extracting (e) The energy barrier of ion diffusion and (f) Desolvation energy.

To evaluate the ion diffusion behavior, EIS was employed on two-electrode supercapacitor cells. In the low-frequency region of the Nyquist plots, the impedance response is dominated by semi-infinite linear diffusion, characterized by the Warburg impedance [64]. The EIS spectra were modeled using an equivalent electrical circuit (Figure 5a). The equivalent circuit comprises an equivalent series resistance (*R*_*s*_) that accounts for the bulk electrolyte resistances, which is determined from the high-frequency intercept with the real impedance axis [65]. This is connected to a parallel combination of the electric double-layer capacitance (*C*) and the charge transfer resistance (*R*_*ct*_). Because charge storage in this system is non-faradaic, *R*_*ct*_ primarily corresponds to the interfacial desolvation resistance, representing the kinetic energy penalty for ions to shed their hydration shells before entering the sub-nanometer pores of the activated carbon [66]. Finally, a finite-length Warburg element (*W*) positioned in series with *R*_*ct*_ models the semi-infinite linear mass diffusion in the low-frequency regime, enabling the extraction of the diffusion resistance and time constant for each temperature [67].

Figures 5b and 5c present the Nyquist plots for the two evaluated electrolytes across a temperature range of 25°C down to −10°C. As the operational temperature decreases, the high-frequency intercept shifts noticeably to the right, indicating an increase in the bulk solution resistance. Concurrently, the slope in the low-frequency region decreases, reflecting more sluggish bulk ion diffusion and heightened mass-transport resistance through the porous electrode at subzero temperatures. To quantify these temperature-dependent kinetic parameters, the experimental Nyquist spectra were modeled using an equivalent electrical circuit in Z-View software, with the extracted values comprehensively summarized in Table S1. Figure 5d provides a direct comparison of the impedance spectra for both electrolytes at 25°C and −10°C. While both systems demonstrate comparable ion transport kinetics at 25°C, their responses diverge significantly under subzero conditions. Specifically, at −10°C, the Nyquist plot for mixed CDHP + CHP (3:1) electrolyte exhibits a distinctly steeper slope in the low-frequency region compared to the pure CDHP electrolyte.

From this fitting process, the diffusion resistance *R*_*W*_ (or W_R_) and the diffusion time constant, *T*_*W*_ (or W_T_), were extracted for each temperature. The Warburg factor (*σ*_*W*_) was subsequently calculated using the established relationship. The method of obtaining Equation 14 is described in method S1 (Equation S3-S8):

(14) $$\sigma_{W}=\frac{R_{W}}{\sqrt{2\cdot T_{W}}}$$

Following standard methodologies for extracting diffusion kinetics from EIS, the absolute ion diffusion coefficient (*D*_*i*_) at each temperature was calculated utilizing Equation 15 [68]: D

(15) $$\frac{0.5\cdot R^{2}\cdot T^{2}}{n^{2}F^{4}A^{2}C^{2}\sigma_{w}^{2}}$$

where *R* is the ideal gas constant, *T* is the absolute temperature, *n* is the number of transferred charges per ion, *F* is the Faraday constant, *A* represents the geometric surface area of the electrode, and *C* denotes the bulk molar concentration of the active electrolyte ions.

The transport of ions through the bulk electrolyte is an important stage in the charge and discharge cycles of a supercapacitor, directly governing its low-temperature power performance. As the operational temperature decreases, the thermal energy available for ion migration diminishes and the viscosity of the aqueous solvent increases, significantly hindering ion mobility. Because the operational temperature critically restricts the thermal energy available for ion migration, the calculated absolute diffusion coefficients were further analyzed using the thermally activated Arrhenius relation (Equation 16) [27].

(16) $$D_{i}=D_{0}\exp\left(-\frac{\Delta G^{\theta }}{\mathrm{RT}}\right)$$

Where *D*_*0*_ is the pre-exponential factor, $\Delta G^{\theta }$ represents the activation energy barrier for bulk ion diffusion, *R* is the ideal gas constant, and *T* is the absolute temperature. To evaluate this, relative diffusion coefficients were derived from the low-frequency Warburg region of the temperature-dependent Nyquist plots, where *D*_*i*_ is inversely proportional to the square of the Warburg factor (*σ*_*W*_). The Arrhenius plots of ln (*D*_*i*_) versus 1000/T (Figure 5e) yielded highly linear fits for both electrolyte systems (*R*^*2*^ > 0.98). Based on the slopes of these linear regressions, the activation energy barrier for ion diffusion ($\Delta G^{\theta }$) is 32.20 kJ ⋅ mol^−1^ for the pure CDHP electrolyte, but it significantly decreases to 27.17 kJ ⋅ mol^−1^ when using the CDHP + CHP mixture. The lower energy barrier in the mixed system means that the ions require less thermal energy to overcome the resistance of the solvent and migrate through the electrolyte matrix. This directly explains why the CDHP + CHP (3:1) electrolyte maintains superior ionic conductivity and higher specific capacitance at subzero temperatures.

The binary salt mixture disrupts the hydrogen bonding network of the water more effectively than the pure salt, preventing the solvent from freezing the ions in place and maintaining faster diffusion kinetics in cold environments. While bulk diffusion dictates how fast ions travel through the electrolyte, the kinetics at the electrode/electrolyte interface are governed by the desolvation process. The solvated shell surrounding the electrolyte ions is typically larger than the sub-nanometer pores of the activated carbon electrodes. Consequently, these ions must partially or fully shed their hydration shells before permeating the pores to form the electrical double layer. The rate of this desolvation process (*k*) is inversely proportional to the interfacial charge transfer resistance (*R*_*ct*_), which corresponds to the diameter of the high-frequency semicircle in the Nyquist plots. The temperature-dependent desolvation kinetics can be modeled using the Arrhenius equation (Equation 17) [69].

(17) $$k=\mathrm{Aexp}\left(-\frac{E_{s}}{\mathrm{RT}}\right)$$

where A is the pre-exponential factor, and *E*_*s*_ is the activation energy required to strip the solvation shell. By plotting ln (1/R_ct_) versus 1000/T (Figure 5f), the interfacial desolvation energy was extracted. For the pure 2 M CDHP electrolyte, the desolvation activation energy was found to be 15.03 kJ ⋅ mol^−1^. Interestingly, for the CDHP + CHP (3:1) mixture, the E_s_ increased to 19.52 kJ ⋅ mol^−1^. This reveals a critical kinetic trade-off inherent to the binary salt system. The introduction of the dibasic hydrogen phosphate anion (HPO_4_^2-^) from the CHP salt presents a higher localized charge density compared to the monobasic dihydrogen phosphate anion (H_2_PO_4_^−^) in pure CDHP. This elevated charge density strengthens the ion-dipole interactions between the phosphate anions and the surrounding water molecules. Consequently, a higher energy penalty is incurred to break these bonds and strip the hydration shell at the electrode interface.

The theoretical framework proposed by Kondrat and Kornyshev [70] establishes that deliberately hindering charge accumulation can paradoxically enhance energy storage by delaying pore saturation. This paradigm is experimentally substantiated by the impedance and desolvation kinetics analyses. Although the CDHP + CHP (3:1) electrolyte facilitates superior bulk ion diffusion, it introduces a substantial kinetic hindrance at the electrode–electrolyte interface. Specifically, the higher localized charge density of the dibasic hydrogen phosphate anion intensifies ion-dipole interactions with the solvent, imposing a significantly elevated interfacial desolvation energy penalty of 19.52 kJ ⋅ mol^−1^, compared to 15.03 kJ ⋅ mol^−1^ for the pure CDHP electrolyte. This increased thermodynamic requirement to strip the hydration shell acts as a deliberate physical resistance, impeding facile ion entry into the sub-nanometer carbon pores at low voltages. By energetically restricting premature electrostatic saturation, this elevated desolvation barrier ultimately enables the sustained accumulation of charge at extended operational potentials.

## 4. Conclusion

This study demonstrates the successful development and electrochemical implementation of a novel, environmentally sustainable aqueous electrolyte based on a binary mixture of CDHP and CHP. While pure CDHP solutions exhibit an acidic pH that inherently restricts the ESW due to the early onset of the hydrogen evolution reaction, the strategic incorporation of the dibasic CHP salt effectively tunes the electrolyte to a near-neutral pH. This neutralization successfully suppresses hydrogen evolution activity, thereby expanding the stable operational voltage of symmetric activated carbon supercapacitors up to 1.5 V, which directly translates into higher energy density.

Furthermore, comprehensive physicochemical and temperature-dependent impedance analyses provided critical kinetic insights into the low-temperature transport mechanisms of these aqueous systems. The CDHP + CHP (3:1) electrolyte demonstrated superior ionic conductivity and higher energy efficiency at subzero temperatures compared to the pure CDHP system. This enhanced cold-weather resilience is attributed to the binary salt mixture effectively disrupting the hydrogen-bonding network of the aqueous solvent, which significantly lowers the activation energy barrier for bulk ion diffusion (to 27.17 kJ ⋅ mol^−1^ from 32.2 kJ ⋅ mol^−1^). However, a fundamental kinetic trade-off was identified at the electrode-electrolyte interface. The introduction of the dibasic hydrogen phosphate anion from the CHP salt presents a higher localized charge density than the monobasic anion in pure CDHP, which strengthens the ion–dipole interactions with surrounding water molecules. Consequently, the mixed electrolyte incurs a higher desolvation energy penalty (19.52 kJ ⋅ mol^−1^ compared to 15.03 kJ ⋅ mol^−1^ for pure CDHP) required to strip the hydration shell before ions can permeate the porous carbon electrodes.

Despite this increased interfacial desolvation resistance, which acts as a deliberate physical resistance to prevent premature electrostatic pore saturation, the synergistic macroscopic benefits of the CDHP + CHP mixture dominate the overall device performance. Ultimately, this work establishes that functionally dynamic, concentrated choline-phosphate mixtures provide a highly viable, low-toxicity, and environmentally sustainable pathway for next-generation advanced electrochemical capacitors operating under broad and extreme temperature conditions.

## Supplementary Material

Supplemental Material

## CRediT author statement

Statement: During the preparation of this work the author(s) used DeepL Translator and GPT-3.5 to find appropriate synonyms, phrases and linking text blocks to better explain the subject and make the text more coherent. After using this tool/service, the author(s) reviewed and edited the content as needed and take(s) full responsibility for the content of the publication.
