# Supplementary material for: Anti-freezing supercapacitors using novel choline phosphate aqueous electrolytes
Source: Sci Technol Adv Mater. 2026 Jun 19;27(1):2688749. doi: 10.1080/14686996.2026.2688749 (PMC13439784; doi:10.1080/14686996.2026.2688749)
Supplement: Supplementary file 1 [file TSTA_A_2688749_SM4921.docx]

**Supporting information**

**Anti-freezing supercapacitors using novel choline phosphate aqueous electrolytes**

Jan Malczak ^a^, Seyed Amirhossein Sanei ^a^, Agnieszka Marcinkowska ^a^, Piotr Gajewski ^a^,

Qamar Abbas ^a, b,*^

*a Faculty of Chemical Technology, Poznań University of Technology, 60-965 Poznań, Poland*

*b Institute for Chemistry and Technology of Materials, Graz University of Technology, 8010 Graz, Austria*

Figure S1. FTIR-ATR spectra of (a) CDHP and (b) CHP salts.

Figure S2. (a) Measured viscosity values at different salt concentrations. As the salt concentration increases, both electrolytes become more viscous. (b) DSC graphs of 2 mol·kg^−1^ CDHP and 3 mol·kg^−1^ CDHP + CHP (3:1) electrolytes.

Figure S3. The conductivity data of the (a) CDHP, and (b) CDHP+CHP (3:1) at different temperatures for concentrations from 1 to 5 M.

Figure S4. (a) Accumulated specific charge evaluated as a function of the applied potential for pure CDHP and mixed CDHP+CHP (3:1) electrolytes at 25 °C and -10 °C, illustrating the temperature-dependent capacitive response and highly linear, ideal electric double-layer charge-storage behavior. (b) Time-dependent current density profiles derived from cyclic voltammetry data, demonstrating the dynamic charging kinetics and highlighting the enhanced high-voltage stability of the mixed CDHP+CHP (3:1) electrolyte, which successfully mitigates the severe leakage current and parasitic hydrogen evolution (HER) observed in the pure CDHP electrolyte near the 1.5 V upper limit.

**Method S1. Calculation of Thermodynamic Hydrogen Evolution Reaction (HER) Potentials**

The thermodynamic onset potential for the hydrogen evolution reaction (HER) is highly dependent on the proton concentration of the aqueous electrolyte. To quantitatively evaluate the effect of pH tuning on the electrochemical stability window, the theoretical reduction potentials for HER were calculated using the Nernst equation at 25$℃$. For the two-electron reduction of protons (2H^+^$\leftrightarrow$ + 2e^-^ +H_2_), the standard Nernst equation simplifies cleanly at standard temperature (298.15 K). By evaluating the universal gas constant (*R*) and Faraday's constant (*F*), and converting the natural logarithm of the proton concentration to a base-10 pH scale, the thermodynamic pre-factor ($\frac{2.303\times R\times T}{F}$) resolves to the constant 0.0591.

| E _HER (vs. SHE)_ = -0.0591 $\times$pH | **Eq. (S1)** |
| --- | --- |

Since the experimental electrochemical measurements were conducted using an Ag/AgCl (2M KCl) reference electrode (where the standard potential E ref$\approx$ +0.212 V vs. SHE at 25 $℃$), the calculated theoretical potentials were converted to the experimental scale using the following relationship to allow for direct comparison with the cyclic voltammetry data:

| E _HER (vs. Ag/AgCl)_ = E _HER (vs. SHE)_ - 0.212 V | **Eq. (S2)** |
| --- | --- |

Applying these equations to the experimentally measured pH values of the prepared electrolytes yields their respective thermodynamic limits. For the pure CDHP electrolyte, with an acidic pH of 4.51, the thermodynamic HER potential is calculated to be -0.478 V vs. Ag/AgCl. Conversely, the introduction of the dibasic salt in the mixed CDHP+CHP electrolyte shifts the pH to a near-neutral value of 6.64, which correspondingly shifts the theoretical HER potential to a more negative value of -0.604 V vs. Ag/AgCl.

Figure S5. Checking HER activity of the electrolytes in negative voltages for (a) CDHP (b) CDHP+CHP (3:1) at 25 $℃$ (c) current density versus time plot for two electrolytes at HER activity voltages.

Figure S6. Specific discharge capacitance at for (a) CHP and (b) CDHP+CHP (3:1).

Figure S7. Dependence of specific energy on temperature for pure CDHP and mixed CDHP+CHP (3:1) electrolytes.

Figure S7 shows that the specific energy of both cells decreased progressively with decreasing temperature. For the CDHP electrolyte, a gradual reduction was observed down to −10 °C, whereas for the mixed CDHP+CHP electrolyte the decrease remained moderate down to −15 °C. At lower temperatures, a much more pronounced deterioration of electrochemical performance was detected, accompanied by a significant loss of capacitance (below 20% of the initial value). This behavior can be attributed to partial electrolyte freezing and the resulting limitation of ion mobility and charge transport processes.

Within the temperature range where both systems remained electrochemically functional (25 to −10 °C for CDHP and 25 to −15 °C for CDHP+CHP), the dependence of specific energy on temperature was approximately linear. Linear regression analysis yielded coefficients of determination of R^2^ = 0.97 and R^2^= 0.94 for the CDHP and CDHP+CHP electrolytes, respectively, confirming a good linear fit to the experimental data. The corresponding regression equations were y = 0.098x + 13.4 for CDHP and y = 0.085x + 10.9 for CDHP+CHP.

| (a) | (b) |
| --- | --- |
|  |  |
| (c) | (d) |
|  |  |

Figure S8. Cyclic voltammetry at: (a) 1.0 V and (b) 1.5 V, GCD performance (c), Nyquist plot (d) results for CDHP+CHP (3:1) electrolyte at −15°C.

Table S2- Extracted fitting parameters from Nyquist plot with fitting of equivalent circuit.

| Electrolyte | Temperature ($℃$) | R_s_ (Ω) | C (μF) | R_ct_ (Ω) | W_R_ (Ω) | W_T_ (s) | σ_W_ (Ω$\cdot$s^-0.5^) | D (s$\cdot$Ω^-0.5^) | Rate constant (S) |
| --- | --- | --- | --- | --- | --- | --- | --- | --- | --- |
| ChH_2_PO_4_ | 25 | 1.12 | 163.11 | 0.34 | 6.43 | 1.49 | 3.73 | 0.07 | 1.09 |
| ChH_2_PO_4_ | 20 | 1.21 | 172.43 | 0.34 | 7.39 | 1.77 | 3.93 | 0.06 | 1.09 |
| ChH_2_PO_4_ | 10 | 1.33 | 177.70 | 0.42 | 10.34 | 2.40 | 4.73 | 0.04 | 0.88 |
| ChH_2_PO_4_ | 0 | 1.58 | 157.53 | 0.53 | 15.40 | 3.37 | 5.93 | 0.03 | 0.63 |
| ChH_2_PO_4_ | -10 | 2.10 | 118.55 | 0.74 | 24.04 | 4.82 | 7.74 | 0.02 | 0.31 |
| ChH_2_PO_4_ and Ch_2_HPO_4_ | 25 | 1.01 | 186.46 | 0.29 | 7.16 | 2.13 | 3.47 | 0.08 | 1.25 |
| ChH_2_PO_4_ and Ch_2_HPO_4_ | 20 | 1.13 | 197.30 | 0.30 | 8.05 | 2.53 | 3.56 | 0.08 | 1.20 |
| ChH_2_PO_4_ and Ch_2_HPO_4_ | 10 | 1.34 | 190.45 | 0.36 | 10.70 | 3.34 | 4.140 | 0.06 | 1.02 |
| ChH_2_PO_4_ and Ch_2_HPO_4_ | 0 | 1.73 | 148.00 | 0.50 | 15.21 | 4.63 | 5.00 | 0.04 | 0.67 |
| ChH_2_PO_4_ and Ch_2_HPO_4_ | -10 | 2.47 | 910.35 | 0.82 | 23.28 | 6.86 | 6.28 | 0.02 | 0.20 |

**Method S2. Derivation of the Semi-Infinite Warburg Coefficient from Finite-Length Diffusion Parameters**

The generalized impedance for one-dimensional, finite-length diffusion can be described by two primary boundary conditions: a transmissive boundary (short-circuit terminus, *Z_W_*_,s_) and a reflective boundary (open-circuit terminus, *Z_W_*_,o_). Their respective impedance expressions are given by [1]:

| Z_W ,s_ = R_w_$\frac{tanh(\sqrt{jwT_{w}})}{\sqrt{jwT_{w}}}$ | **Eq. (S3)** |
| --- | --- |
| Z_W ,o_ = R_w_$\frac{coth(\sqrt{jwT_{w}})}{\sqrt{jwT_{w}}}$ | **Eq. (S4)** |

where *R_w_* is the low-frequency diffusion resistance, *T_w_* is the diffusion time constant (T_w_ = L^2^/D), *j* is the imaginary unit, and ω is the angular frequency. At sufficiently high frequencies (ω$\to\infty$), the AC perturbation oscillates rapidly enough that the diffusion layer thickness is significantly smaller than the physical boundary length (*L*). Under this condition, the system exhibits semi-infinite diffusion behavior. Mathematically, as ω$\to\infty$, the hyperbolic terms converge to unity (tanh($\infty$)$\approx$1 and coth($\infty$)$\approx$1). Consequently, both finite-length models reduce to the same high-frequency asymptote:

| Z_W_$\approx\frac{R_{w}}{\sqrt{jwT_{w}}}$ | **Eq. (S5)** |
| --- | --- |

To separate this expression into its real and imaginary components, we apply the complex identity $\frac{1}{\sqrt{j}}=\frac{1-j}{\sqrt{2}}$.

| Z_w_$\approx\frac{R_{w}}{\sqrt{wT_{w}}}$ ($\frac{1-j}{\sqrt{2}}$) = $\frac{R_{w}}{\sqrt{2wT_{w}}}(1-j)$ | **Eq. (S6)** |
| --- | --- |

This asymptotic limit is definitionally equivalent to the classical model for semi-infinite linear diffusion (the standard Warburg impedance), which is expressed as:

| Z_semi_= $\frac{\sigma_{w}}{\sqrt{w}} (1-j)$ | **Eq. (S7)** |
| --- | --- |

where σ_w_ is the Warburg coefficient. By equating the magnitude of the real (or imaginary) components of the two asymptotic expressions, the relationship between the macroscopic finite-length parameters and the intrinsic Warburg coefficient is established:

| $\frac{\sigma_{w}}{\sqrt{w}}$=$\frac{R_{w}}{\sqrt{2wT_{W}}}$ | **Eq. (S8)** |
| --- | --- |

**The effect of viscosity on the energy performance of the symmetric cell**

As demonstrated by the physicochemical characterizations, the mixed CDHP + CHP electrolyte exhibits a higher viscosity compared to the pure CDHP electrolyte. Thus, a direct result of these restricted ion transport kinetics is that the supercapacitor utilizing the mixed electrolyte system experiences a relative reduction in both overall energy density and energy efficiency when compared to the less viscous, pure CDHP system.

**References**

[1]	S. Cruz-Manzo and P. Greenwood, "Analytical transfer function to simulate the dynamic response of the finite-length Warburg impedance in the time-domain," *Journal of Energy Storage,* vol. 55, p. 105529, 2022.
